# Can Students Create Their Own Educational Escape Room? Lessons Learned from the Opioid Crisis Escape Room

**DOI:** 10.1007/s40670-021-01425-5

**Published:** 2021-10-15

**Authors:** Michiel J. Bakkum, Milan C. Richir, Rowan Sultan, Jara R. de la Court, Anke C. Lambooij, Michiel A. van Agtmael, Jelle Tichelaar

**Affiliations:** 1 Department of Internal Medicine, Section Pharmacotherapy, Amsterdam UMC, Vrije Universiteit Amsterdam, De Boelelaan 1117, 1081 HV Amsterdam, Netherlands; 2 Research and Expertise Centre in Pharmacotherapy Education (RECIPE), De Boelelaan 1117, 1081 HV Amsterdam, The Netherlands; 3 Dutch Institute For Rational Use of Medicines (IVM), Churchilllaan 11, 3527 GV Utrecht, The Netherlands; 4 https://www.eacpt.eu/

**Keywords:** Clinical pharmacology and therapeutics, Serious gaming, Educational escape room

## Abstract

Educational escape rooms (EERs) are live-action, team-based games used to teach content-related and generic knowledge and skills. Instead of students just playing the EER, we believed that giving them the opportunity to create their own EERs would augment the learning effects of this teaching method. We report on the feasibility, evaluation, and lessons learned of our assignment on an opioid epidemic-based EER. This original teaching method appealed to most students, but the workload was evaluated to be too high. Our lessons learned include the need for sufficient (extrinsic) motivation, careful explanation of the assignment, and small group sizes.

## Background

The prescription and misuse of opioids has become a problem in many countries [1, 2]. In order to mitigate opioid abuse, we thought it important to teach students about the dangers of opioid dependency and the important preventive role of prescribers. Given the topicality and international relevance of the subject, we aimed to create an engaging and widely usable learning experience for undergraduate and graduate medical students, nurses, non-medical prescribers etc. that could be easily shared (as open educational resource) with colleagues in the international network of teachers in pharmacotherapy via the EurOP^2^E platform (www.prescribingeducation.eu).

Escape rooms are live-action, team-based games in which players discover clues, solve puzzles, and accomplish tasks in one or more rooms in order to achieve a specific goal (usually escaping from the room) in a limited amount of time [3]. There are more than 10,000 recreational escape rooms worldwide [4]. The success of these games has prompted teachers in all levels and types of education, including medical education, to recreate these games for educational purposes [5]. Educational escape rooms (EERs) are based on experiential, game-based, and team-based learning principles to increase the intrinsic motivation to learn. This is best explained by the self-determination theory (SDT), which states that humans are self-determined to grow and learn and that this growth is fostered by fulfilling three psychological needs: autonomy, competence, and relatedness [6]. Gamified, team-based learning methods, such as EERs are attractive because they allow students to satisfy these three needs — autonomy via freedom of choice (e.g., to approach puzzles in multiple ways), competency via direct feedback on the progress (e.g., locks that open), and relatedness via a common cause (to escape the room) that requires teamwork [7]. A greater intrinsic motivation is associated with better learning and conceptual understanding [6].

The use of EERs in medical and pharmacy education is rising for a variety of purposes [7], ranging from team-building exercises [8] and research [9] to learning and assessing knowledge, skills, and attitudes [10–12]. A recent review found that while EERs appeal to students, helping them to consolidate existing knowledge and skills, they appear to be less effective for teaching new concepts [13]. Another often reported drawback of EERs is that they are challenging and labor-intensive to produce. In an international survey among creators of recreational escape rooms (*n* = 175), almost 50% found creating puzzles and balancing the difficulty of puzzles to be “very challenging” [3], as is the case for EERs [14]. We believed that we could use these apparent drawbacks of EERs to our advantage. Asking the students to create the tasks and puzzles for their own EER would provide them with a challenging assignment that could improve the learning effects while possibly limiting the time commitment of teaching staff. According to the SDT principles, we aimed to give the students full autonomy in the development of the puzzles, make this a team exercise, and enhance the purpose of this assignment beyond that of a single lesson. Therefore, we implemented the following interrelated aspects that were previously shown to enhance motivation in student-led education (SLE): an authentic context with authentic responsibility (to create a real educational resource for use in national and international pharmacotherapy education) and collaboration between students and professionals [15, 16]. This is alike our previous and ongoing student-led education (SLE) projects, wherein students run several outpatient clinics [17, 18], a pharmacovigilance program [19], and a medication review team [20] — all with supervision but full responsibility for the actual care of patients. These SLE projects were shown to have great learning effects, as well as clinical results and consistently very good evaluations [17–21].

With the goal to inform and inspire teachers, this article describes the pedagogical background, feasibility, implementation, evaluation, and lessons learned from our assignment for year 3 medical students at the Amsterdam University Medical Center to create an EER about the opioid epidemic.

## Activity

### Learning Goals

Coinciding with the development of the EER, the Dutch Ministry of Health started a program to reduce inappropriate use of opioids [22]. This program is led by the Dutch Institute for Rational Use of Medicines (IVM) in close collaboration with scientific societies of prescribers and pharmacists. With the aim of creating a uniformly usable EER, we collaborated with the IVM to establish learning goals for the EER (and the assignment to create the EER), namely, (1) recognizing and treating opioid addiction, (2) preventing legal and illegal access to prescription opioids, (3) patient education on safe use of opioids, (4) treating a patient with an acute opioid overdose, and (5) safe and effective prescribing of opioids.

### General Design of the EER

**Fig. 1:**
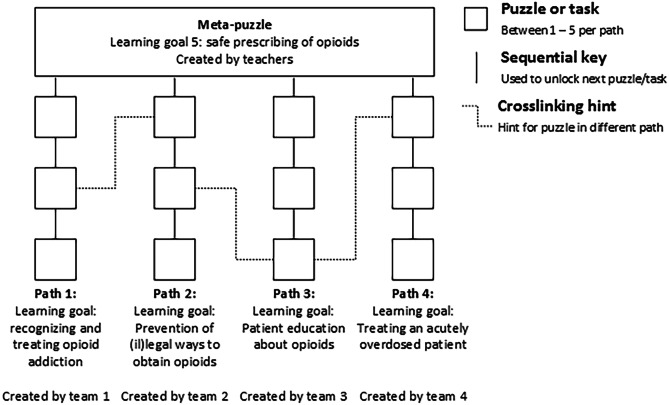
Design schematic. The puzzle was path-based according to the definition by Nicholson, meaning that several (simultaneously playable) sets of puzzles and tasks (“paths”) would provide essential clues for the final (“meta”) puzzle^3^. Each of the paths and the meta-puzzle were associated with one of the learning goals

The EER was path-based according to the definition by Nicholson [3], meaning that several (simultaneously playable) sets of puzzles and tasks (“paths”) provided essential clues for the final (“meta”) puzzle (Fig. 1). Only by completing all the puzzle paths, the players received sufficient information to solve the meta-puzzle and then escape the room. In line with the intrinsic integration theory of van der Linden et al. [23], the paths and meta-puzzle each taught information related to one of the learning goals. In a path-based EER, the most effective strategy to escape the room is to divide the work (e.g., student A tries to solve path 1 while student B works on path 2); therefore, a drawback of this design is that individual students do not play all the paths (and learn about all learning goals).

### The Assignment to Create the EER

Thirty-nine year 3 (pre-clinical, bachelor) medical students, who were taking an elective course on internal medicine at the Amsterdam Medical Center, VU University, were (alphabetically) divided into three groups (*n* = 13). Each group was asked to make a prototype EER. The assignment was scheduled over a 4-week period and started with a 15-min plenary briefing during which the students received information about the general design of the EER (see above) and plan for the assignment. The groups of students were subdivided into four teams (3–4 students), and each team was given the task to create one of the paths (Fig. 1). The teachers (M.B. and J.C., two junior doctors who are Ph.D. students and teachers in clinical pharmacology) developed the meta-puzzles (one for each prototype EER). During the 4 weeks, students could schedule guidance sessions with one of the teachers, for example to discuss whether a task would be too difficult or if they could use specific items (ranging from simple office supplies to food dye to be used in IV-bags). The teachers forbade nothing but sometimes motivated students to come up with more creative puzzles or more practical solutions. On account of the late deadline (day before playing the rooms), the teachers were unable to check the puzzles for inaccuracies. Teams were free to shape their paths, for example they could create one large puzzle or a sequence of multiple smaller puzzles — the only requirement was that the path could be solved in approximately 10 min. Students were encouraged to create cross-linking hints with other teams in their group (Fig. 1), meaning that one team’s puzzle would provide a (non-essential but helpful) clue towards solving the other team’s path. The students were free to decide how to give substance to the learning goals and could use any trustworthy source of information at their disposal. In terms of difficulty, the students were told that the ability to progress in the puzzle path should not be dependent on prior knowledge or experience, but rather on the ability to deduce or find essential information. This was done to make the EER fun and challenging for a wide audience with various backgrounds and levels of education. Lastly, because we wanted the game to be portable and easy to share internationally, students could only use small, every-day, and preferably digital materials (such as padlocks and QR-codes). They were not given a fixed budget but could submit a shopping list of reasonably priced materials for the teachers to acquire. To further increase student motivation, a professional jury (with the dean of medicine and a representative from the IVM) was selected, and the team with the most creative, challenging, and educational path was given a visit to a recreational escape room as prize. Examples and a video-impression of the assignment can be viewed at http://www.prescribingeducation.eu/opioid-crisis-escape-room

### Playing the EERs

On the last day of the assignment, the prototypes were set up in three separate classrooms. The groups of students rotated the classrooms where they played and tested the EERs. Each rotation lasted 40 min, 30 min to escape the room and 10 min to reset the puzzles and have short debrief. In their “own” room, students did not play (as they knew the answers). Instead, they explained their path to the jurors. Before playing the EER, most chairs and tables were set aside, and the puzzles were installed across the room by the teachers. Items such as clocks and bins were left in place and at times used as hiding places for parts of a puzzle. No “red herrings” (false clues or objects with the purpose to distract) were used. The EERs were themed around the (rather spooky) “Poppy Fields Nursing Home,” and the task of the meta-puzzle was to prescribe the correct treatment (suitable alternative to an opioid analgesic) for the right patient before time ran out. The four puzzle paths revealed which patient was to be treated, a list of patients’ problems, the current medication of the patient, and a (mock) treatment guideline — all of this information was necessary to solve the meta puzzle. The prescription had to be entered in a javascript-based computer program, which validated the answer and showed a congratulatory message with the time it took to “escape” the room. The door was never actually locked. Other than the slightly decorated puzzle items, no decorations were used to make the classrooms look like a nursing home. The EERs were played by a full group (*n* = 13) of students at a time. Cellphones were allowed for specific purposes only, for example to scan QR codes or to send or receive clues via text message. The teacher monitored time and provided hints as necessary. For some puzzle-paths, teachers were required to act as game masters, for example, asking questions in a quiz-like setting.

### Ethical Considerations

Playing and creating the room were ungraded assignments, and the opioid-themed learning goals were not part of the final exam. The assignment was part of the normal curriculum of the elective course, and under Dutch law, the evaluation does not fall within the Medical Research Involving Humans Act (WMO); therefore, no ethical approval was required. Participation in the assignment was compulsory, but the evaluation was voluntary. Students who appear in the promotional video provided informed consent to our communications department.

## Results

### Students’ Evaluations

Thirty-eight students answered the anonymous voluntary survey about playing the EER; 2 failed to complete the survey about creating the room. Results for the 5-point Likert-type questions about *creating* the escape room are shown in Table 1. A total of 47.2% of students agreed (or completely agreed) that they liked this assignment, and 44.4% (completely) agreed that they learned a lot. A total of 56.9% (completely) agreed that the assignment was too much work; only 31.9% did not mind this because of the learning effects. Recurring themes on the open-ended question “what did you learn from *creating* the room?” were opioid-related learning goals (e.g., “The importance of preventing an opioid crisis, treatment of acute overdoses, and rehab medicine”, 7 times), creative thinking (4 times), and teamwork (2 times). Freedom and creative thinking were the most (10 times) appreciated aspects of the assignment, followed by creating a puzzle (4 times). On the last question “how could we improve the assignment?” 8 students answered that clearer instructions should be provided, 4 that too little time was available, 2 that the overall workload was too high, and 2 that the timing in relation to other assignments was poor. While only 1 student made a written comment, we heard that students were not highly motivated because the assignment was not graded.

**Table 1:** Students’ evaluations about *creating* the EER (*n* = 36)

	**Completely disagree (%)**	**Disagree (%)**	**Neutral (%)**	**Agree (%)**	**Completely Agree (%)**
I liked creating the EER	–	16.7	36.1	27.8	19.4
I learned a lot from creating the EER	2.8	33.3	19.4	36.1	8.3
The opioid-related learning goals are now clear to me	–	6.9	34.7	5–	8.3
The assignment is better than a traditional lecture or practical	2.8	15.3	25.0	37.5	19.4
Creating the EER was too much work	–	27.8	15.3	45.8	11.1
I did not mind the work, because I learned a lot	8.3	38.9	20.8	20.8	11.1
The assignment was clearly explained	2.8	19.4	44.4	30.6	2.8
It was nice to have support from the teacher	–	–	25.0	5–	25.0
The quality of the support was good	–	11.4	25.7	51.4	11.4
Next year, the course should have a similar assignment	5.6	19.4	25.0	25.0	25.0
The plan to use my work in an international EER was motivating	2.8	16.7	13.9	30.6	36.1
Creating the EER was one of the better assignments in the course	2.8	19.4	30.6	30.6	16.7

36 students answered questions about creating the EER, 38 about playing the EER. When two adjacent answers were provided for one statement (e.g., neutral and agree), each was counted as half

*EER* educational escape room

Results for the 5-point Likert-type questions about *playing* the escape room are shown in Table 2. A total of 80.3% agreed (or completely agreed) that they liked playing the escape room, and 26.3% agreed that they learned a lot from playing the room. Of the students, 67.1% believed the EERs to be a little too difficult. The majority of students (60.5%) believed that the rooms were suited for 7–9 players at a time. On the questions “what did you learn from *playing* the room?” 12 students answered teamwork, 10 opioid-related learning goals, and 2 performing under pressure; however, 4 said they had learned “nothing” or “too little,” with explanation that the group was too large. Aspects students liked about playing the room were the actual puzzles (8 times), teamwork and brainstorming together (6 times), and the creative and original teaching method (4 times). On the question “what can we improve?” 11 students answered problems with specific puzzles, such as them being too difficult, too basic (simple crosswords), or containing errors. Four students answered that smaller groups would improve the experience. Lastly, 4 students disliked how independent the four puzzle paths were and suggested improving connections between these paths.

**Table 2:** Students’ evaluations about *playing* the EER (*n* = 38)

	**Completely disagree (%)**	**Disagree (%)**	**Neutral (%)**	**Agree (%)**	**Completely Agree (%)**
I liked playing the EER	–	–	19.7	59.2	21.1
I learned a lot from playing the EER	–	30.3	43.4	22.4	3.9
The opioid-related learning goals are now clear to me	2.7	18.9	37.8	36.5	4.1
Playing the EER is better than a traditional lecture or practical	2.6	18.4	31.6	28.9	18.4
The students’ puzzles were of sufficient quality	5.3	5.3	43.4	38.2	7.9
The teachers’ puzzles were of sufficient quality	–	–	10.5	57.9	31.6
Next year, the course should have an EER to play	–	10.5	28.9	34.2	26.3
Playing the EER was one of the better assignments in the course	5.3	15.8	22.4	25.0	31.6
	**Too easy (%)**	**A little too easy (%)**	**Right difficulty (%)**	**A little too difficult (%)**	**Too difficult (%)**
How difficult was playing the EER?	–	–	26.3	67.1	6.6
	**5 or less players (%)**	**5–6 players (%)**	**7–8 players (%)**	**8–9 players (%)**	**10 or more players (%)**
How many players can the rooms accommodate?	–	23.7	31.6	28.9	15.8

## Discussion

Overall, this invention provided a fun, interactive, and stimulating way to teach students about the dangers of prescription opioid abuse and how to prevent it. The jurors were impressed by the engaging puzzles the students had created, some of them not unlike those in recreational escape rooms. These puzzles provide a strong foundation for the EER that we aim to further develop with the students, for use in national and international education. The students’ evaluations, however, were not uniformly positive, leaving room for improvement for future assignments and for anyone aiming to implement a comparable teaching method.

First, we had overestimated the motivation of the students in relation to the workload. We expected the fun, creative, and original method of learning would be motivating in itself, combined with the competitive elements and the fact that the students would be contributing to a lasting, internationally used educational experience. While almost two-thirds of students agreed that the international plans for the EER helped motivate them, not all the students seemed to appreciate the potential impact of their work. We even overheard them discussing how the teachers were using them to do their (the teachers’) work. This feedback surprised us, because it has never been mentioned in our student-run clinics, which also rely heavily on context-based learning with real responsibilities [15, 16]. Two important differences may explain this discrepancy. First, for medical students, treating a patient is probably a context they can relate to, in contrast to creating an educational experience. Secondly, the clinics are run by students who are actively looking for learning opportunities, either during their clinical rotations or because they have signed up voluntarily. However, this assignment was compulsory, which possibly explains why students found the workload high and poorly planned in relation to other, graded, learning activities. We believe that motivation, and not the actual limited workload of 8–12 h, was the problem. We suggest that, in the future, the assignment should be graded or that its learning objectives should be constructively aligned with a test.

Secondly, given the problem of motivation, it is not surprising that some students put minimal effort into the assignment, handing in basic crosswords and unclear or even unsolvable puzzles. This directly affected the learning experience of other students, who were annoyed by the poor design and lack of progress. Students also disliked the lack of cohesion between the different paths. While we aimed to improve this by introducing cross-linking hints, the assignment was difficult to properly explain and, in hindsight, it was insufficiently understood by the students.

Lastly, due to a fixed schedule, we were forced to play and test the EERs with relatively large groups of 13 students. This meant that individual students played less than half of the available puzzles, reducing the experience and learning effects of the EER. The majority of students believed that the EERs were suitable for maximally 7–9 players.

From a teacher’s perspective, the assignment was also challenging. Tutoring the teams, getting their shopping lists in time, buying the diverse items on these lists, properly understanding all paths, joining them together in playable prototypes, and creating the meta-puzzles were all time-consuming (estimated to be at least two full-time weeks for a single teacher) and at times stressful activities. That said, the teachers found it very fulfilling that three playable prototypes were created.

While the majority of students liked playing the EER, they were less positive about the educational yield of this part of the assignment. One of the biggest challenges of creating EERs is to balance the learning effects with fun. Puzzles that are packed with content are often quite dull (e.g., a quiz-like puzzle or crossword), whereas more creative puzzles tend to lose learning effects (e.g., time spent counting green and blue pills, is time not spent on the opioid-related learning goals). Both the students and teachers had difficulties to find this balance, and we believe that all puzzles need improvement and further testing before they can be used in the final EER. The limited educational yield, however, is a common critique of EERs [13, 14]. In order to create the puzzles, students need a deeper understanding of the learning goals. Indeed, the students evaluated the learning effects of creating the room more positively. Therefore, we believe that adding the assignment to create the EER is a promising way to increment the learning effects of an EER while maintaining the positive effects of (playing) EERs.

This article shows the feasibility of having students create the tasks and puzzles for an EER. This novel teaching method was well-evaluated and improved the student-reported learning effects toward our opioid-related learning goals as compared to playing the EER. With minor modifications (see lessons learned), we believe that this method could provide a valuable addition to the teaching repertoire of healthcare teachers.

### Limitations

Investigating the learning effects of creating the EER in an objective (i.e., more than student-reported) manner was beyond the scope of this project and should be subject to further investigation.

## Lessons Learned

To our knowledge, we are the first to publish about this promising teaching method. With the aim to assist teachers elsewhere in creating similar assignments, we have summarized our lessons learned:

1. Learning objectives for the assignment should have an authentic and relatable context.
2. Learning objectives should be paired with puzzle paths; limiting the number of paths may help to unite the puzzles into an EER
3. It should be clear to the students why they are asked to create the puzzles and paths. That is to enhance the educational effects via peer-teaching
4. The assignment should be properly explained and include background information on the effects and design of EERs, preferably with examples of educational puzzles
5. The assignment should be constructively aligned in the course. Grading the activity could help to improve student motivation, especially if participation is compulsory
6. Costs for materials can be kept low. However, the assignment may become costly in terms of man-hours
7. Sufficient time should be given for the students to create the paths; early deadlines and sufficient coaching may help to avoid last-minute stress
8. Team sizes during creating and playing the EERs should be kept small. This may make it logistically challenging to accommodate large numbers of students
